# Postexercise hypotension and related hemodynamic responses to cycling under heat stress in untrained men with elevated blood pressure

**DOI:** 10.1007/s00421-020-04340-6

**Published:** 2020-03-18

**Authors:** Felipe A. Cunha, Paulo Farinatti, Helen Jones, Adrian W. Midgley

**Affiliations:** 1 Postgraduate Program in Exercise Science and Sports, University of Rio de Janeiro State, Rio de Janeiro, Brazil; 2 Laboratory of Physical Activity and Health Promotion, University of Rio de Janeiro State, Rio de Janeiro, Brazil; 3 Post-Graduate Program in Physical Activity Sciences, Salgado de Oliveira University, Niterói, Rio de Janeiro, Brazil; 4 Research Institute for Sport and Exercise Sciences, Liverpool John Moores University, Liverpool, UK; 5 Department of Sport and Physical Activity, Edge Hill University, Ormskirk, L39 4QP Lancashire UK; 6 Postgraduate Medical Institute, Edge Hill University, Ormskirk, Lancashire UK

**Keywords:** Baroreceptor reflex, Blood pressure, Cardiovascular response, Energy expenditure, Thermoregulation

## Abstract

**Purpose:**

To investigate the effect of heat stress on postexercise hypotension.

**Methods:**

Seven untrained men, aged 21–33 years, performed two cycling bouts at 60% of oxygen uptake reserve expending 300 kcal in environmental temperatures of 21 °C (TEMP) and 35 °C (HOT) in a randomized, counter-balanced order. Physiological responses were monitored for 10-min before and 60-min after each exercise bout, and after a non-exercise control session (CON). Blood pressure (BP) also was measured during the subsequent 21-h recovery period.

**Results:**

Compared to CON, systolic, and diastolic BPs were significantly reduced in HOT (Δ = − 8.3 ± 1.6 and − 9.7 ± 1.4 mmHg, *P* < 0.01) and TEMP (Δ = − 4.9 ± 2.1 and − 4.5 ± 0.9 mmHg, *P* < 0.05) during the first 60 min of postexercise recovery. Compared to TEMP, rectal temperature was 0.6 °C higher (*P* = 0.001), mean skin temperature was 1.8 °C higher (*P* = 0.013), and plasma volume (PV) was 2.6 percentage points lower (*P* = 0.005) in HOT. During the subsequent 21-h recovery period systolic BP was 4.2 mmHg lower in HOT compared to CON (*P* = 0.016) and 2.5 mmHg lower in HOT compared to TEMP (*P* = 0.039).

**Conclusion:**

Exercise in the heat increases the hypotensive effects of exercise for at least 22 h in untrained men with elevated blood pressure. Our findings indicate that augmented core and skin temperatures and decreased PV are the main hemodynamic mechanisms underlying a reduction in BP after exercise performed under heat stress.

## Introduction

A fall in blood pressure is commonly observed after performing a single bout of aerobic exercise, which can persist for up to 24 h when compared to the immediate pre-exercise period (baseline), or a non-exercise control day (Pescatello et al. 2004). The magnitude and duration of this phenomenon, known as postexercise hypotension (PEH), has been suggested to predict the long-term reduction in blood pressure elicited by aerobic training in prehypertensive individuals (Liu et al. 2011), and depends on several factors. These factors include the acute training variables used for exercise prescription, such as intensity, duration, and mode of activity, as well as baseline blood pressure, with progressively more marked hypotensive responses in hypertensive individuals compared to normotensives (see Pescatello 2015 for a review). Less attention has been given to the influence of environmental conditions (Franklin et al. 1993; Lynn et al. 2009). PEH is a highly regulated physiological phenomenon where mean arterial pressure (MAP) is the result of the relationship between cardiac output (Q) and systemic vascular resistance (SVR), via cardiac (central) and vascular (peripheral) function. These mechanisms are affected by heat stress during exercise performed in hot environments (Gonzalez-Alonso et al. 2008; Periard et al. 2011; Wingo et al. 2005) and, therefore, it is feasible that acute blood pressure responses to aerobic exercise are influenced by environmental temperature.

Although aerobic exercise has consistently been shown to induce hypotensive effects in the postexercise period, the underlying physiological mechanisms are still unclear, especially when the exercise is performed in a hot environment. In the first study investigating this topic, involving 11 trained normotensive men, Franklin et al. (1993) evaluated the effect of thermoregulatory mechanisms on PEH during 60-min of recovery from 30-min of upright cycling exercise at 70% VO_2max_ under cool (17.0 ± 0.8 °C and 57.8 ± 4.3%), neutral (21.4 ± 0.5 °C and 51.8 ± 4.6%), and warm (31.1 ± 0.4 °C and 53.0 ± 4.6%) environmental conditions. Compared to pre-exercise, PEH was only evident when recovery took place in the warm environment [∆ systolic blood pressure (SBP) and MAP: − 6.5 and − 4.7 mmHg, respectively], which also was associated with significantly higher mean skin temperature (MST) and core temperature (*T*_c_). From a practical perspective, this finding suggests that cutaneous vasodilation may have a key role in inducing a greater magnitude and duration of PEH. Notably, however, this study did not assess physiological variables that have been consistently associated with changes in PEH, such as Q, stroke volume (SV), SVR, plasma volume (PV), and cardiac autonomic function [i.e. baroreflex sensitivity (BRS) and heart rate variability (HVR)] (Cunha et al. 2015b; Halliwill et al. 2013). Another limitation is that ambulatory blood pressure monitoring (ABPM) to establish the duration of the effects of heat stress on PEH was not used. Furthermore, the isolated effect of heat stress during exercise on subsequent PEH was not investigated, since all the exercise bouts were performed in a thermoneutral condition (20.8 ± 0.8 °C and 57.3 ± 5.9%) and exposure to different environmental temperatures only occurred postexercise (Franklin et al. 1993). Previous research on this topic (Franklin et al. 1993; Lynn et al. 2009) also did not compare the hypotensive responses in untrained individuals with elevated blood pressure. In contrast to a reduced Q in endurance trained populations (Dujic et al. 2006; Senitko et al. 2002), it has been suggested that PEH is secondary to decreased SVR in the general population (Brito et al. 2014; Halliwill et al. 2013). Therefore, the methodological issues associated with previous research make the underlying physiological mechanisms underpinning PEH, and its clinical relevance, unclear within the context of aerobic exercise prescription under heat stress conditions in non-athletic populations.

To promote health among adults, the American College of Sports Medicine (ACSM) recommends a target energy expenditure of between 150 and 400 kcal per exercise bout and an exercise intensity of between 40 and 85% heart rate reserve (HRR) or oxygen uptake reserve (VO_2_R), assuming a 1:1 relationship between the %HRR and %VO_2_R (see da Cunha et al. 2011b for a review). From a practical perspective, the HR can be used to monitor and adjust power output (usually determined from the ACSM metabolic equation for cycling) to achieve the target metabolic intensity, while the VO_2_ can be used to determine the duration of exercise required to elicit a target energy expenditure. However, whether the change in HR is a valid marker of change in metabolic intensity during prolonged exercise in hot environments is uncertain. The study of Wingo et al. (2005) observed that between 15 and 45 min of constant-load cycling at ~ 63% VO_2max_ in a hot environment (35 °C), HR increased by 12% (151 ± 9 vs. 169 ± 10 beats/min, *P* < 0.05), whereas VO_2_ increased only by 1.9% (2.69 ± 0.19 vs. 2.74 ± 0.19 L/min, *P* < 0.05). Based upon the maximal HR and VO_2_ values obtained during a maximal cardiopulmonary exercise test (CPET) in a temperate environment, the absolute changes in HR and VO_2_ revealed a non-linear relationship between %HRR vs. %VO_2_R (15 min: 69.0 ± 5.5% vs. 60.2 ± 4.1%, respectively; 45 min: 84.0 ± 6.0 vs. 61.2 ± 3.8, respectively). Likewise, those authors also found that constant‐load cycling in the heat was associated with a decrease in VO_2max_ and an increased %VO_2_R. Indeed, the findings of previous studies have indicated marked reductions (e.g. 16–25% or 750–985 mL/min) in VO_2max_ during heat stress, when *T*_c_ was elevated before the CPET (Nybo et al. 2001; Pirnay et al. 1970). It is clear that prescribing exercise intensity based upon the %HRR response and then estimating the metabolic intensity from %VO_2_R assuming a 1:1 relationship, would probably overestimate energy expenditure, especially within the context of prolonged exercise bouts performed under heat stress conditions. On the other hand, the exercise pressor response, which seems to influence blood pressure after exercise, has been shown to be affected by the total amount of muscle work performed and is a surrogate of exercise volume (Chen and Bonham 2010; Halliwill et al. 2013). A recent study by Cunha et al. (2015b), for example, investigated the effects of different CPET modalities (i.e. matched by the maximal intensity) on the magnitude of PEH in healthy men, and observed that only running (exercise mode involving greater energy expenditure) was able to induce PEH. No previous research investigating the effects of heat stress on PEH prescribed exercise bouts by assessing the VO_2_ and energy expenditure and, instead, used HR or a measure of external work rate associated with VO_2max_ as indicators of metabolic intensity. Hence the exercise volume performed has not been properly controlled, which is an important methodological issue that may confound the interpretation of the effects of heat stress on the magnitude of PEH.

Further research is clearly warranted to investigate PEH elicited by exercise bouts performed in a hot versus temperate environment. The mechanistic basis of PEH also needs to be better understood. Thus, the main purpose of the present study was to evaluate the effect of cycling bouts, performed in a hot versus temperate environment, on PEH in untrained men with elevated blood pressure during a 22-h recovery period. We hypothesized that exercise bouts performed in different environment temperatures would elicit different magnitudes and durations of PEH. A second aim was to investigate the physiological mechanisms underpinning any differences in PEH in response to changes in environmental temperature during the first hour of recovery.

## Methods

### Ethical Approval

Subjects were informed of the requirements, benefits, and potential risks and discomforts prior to the commencement of the study, and subsequently gave written informed consent. The experimental procedures adhered to the ethical guidelines outlined in the Declaration of Helsinki and the study gained approval from the Human Research Ethics Committee at Edge Hill University. All subjects were informed of the potential risks and discomforts prior to the commencement of the study, and subsequently gave written informed consent.

### Participants

Seven untrained men volunteered for the study [mean (SD), age: 26.0 (4.8) years; height: 173.9 (4.3) cm; body mass: 76.8 (7.7) kg; body mass index: 25.7 (0.9) kg m^2^; SBP: 127.4 (5.9) mmHg; diastolic blood pressure (DBP): 83.7 (3.0) mmHg; resting heart rate: 60 (10) beats/min; resting VO_2_: 0.24 (0.03) L/min; maximal heart rate: 182 (5) beats/min; maximal VO_2_: 2.80 (0.66) L/min]. Inclusion criteria dictated that participants were men who were deemed inactive according to the definition of the American College of Sports Medicine [i.e. not participating in ≥ 30 min of moderate-intensity physical activity on ≥ 3 days per week for ≥ 3 months prior to the study (ACSM 2018)], with a mean screening SBP of 120–139 mmHg and/or diastolic blood pressure (DBP) of 80–89 mmHg (Whelton et al. 2018). The following exclusion criteria were applied: (a) use of cardiovascular and/or metabolic medications; (b) smoking or use of other tobacco products; (c) use of nutritional ergogenic substances that could affect exercise performance; (d) diagnosis of any cardiovascular, respiratory, bone, muscle, or joint problems; and (e) SBP < 120 mmHg and DBP < 80 mmHg.

Blood pressure screening followed European Society of Hypertension guidelines (Topouchian et al. 2006). Participants remained seated in an upright position in a quiet and comfortable environment to stabilize blood pressure, with the right arm resting on a table at heart level. After 15-min of seated rest, three assessments were performed interspersed by 3-min intervals, using an oscillometric device (OMRON™—HEM-433 INT, Bannockburn, IL, USA). Blood pressure was recorded as the mean of the three readings.

### Experimental design

Each subject visited the laboratory on five occasions to undertake the following procedures: Visit 1: complete a pre-exercise medical questionnaire, blood pressure screening to ensure the study inclusion criteria were met, and anthropometric measurements; Visit 2: perform a resting VO_2_ assessment and CPET in a temperate environment (TEMP, 21 °C); and Visits 3–5: perform a non-exercise control session (CON) in TEMP, and two exercise bouts expending 300 kcal at 60% VO_2_R in TEMP and hot (HOT, 35 °C) environmental conditions. The predicted time to achieve 300 kcal during the cycling bouts ranged from 1860 to 3695 s, which was calculated individually from the net VO_2_, assuming that the consumption of 1 L of oxygen releases approximately 5 kcal of energy. All the experimental procedures were performed in an environmental chamber (Grant Instruments, Cambridge, UK) with a constant relative humidity of 40%. The experimental trials were separated by 24–48 h and performed in a randomized, counterbalanced manner.

Prior to undertaking the experimental trials, participants were given 300 mL of water to drink to promote hydration and then body mass was measured. During the baseline and recovery periods associated with CON, TEMP and HOT, physiological measurements were obtained in a supine position for 10-min and 60-min, respectively; however, for the recovery period, the room was kept at a relatively constant temperature (TEMP, 21 °C). When participants achieved 300 kcal in HOT, both doors of the chamber were immediately opened and the thermostat was set to TEMP condition within 10 s of exercise termination. All visits were conducted at the same time of day, starting at 10:00 a.m., to negate any effects of circadian variation. Participants were required to abstain from physical exercise, alcohol, soft drinks, and caffeine in the 24 h preceding the assessments, and fast for 3 h preceding the assessments.

### *Resting VO*_*2*_* assessment*

Resting VO_2_ was determined prior to calculation of the %VO_2_R in accordance with the recommendations of Compher et al. (2006). In brief, this consisted of abstention of physical exercise, alcohol, soft drinks, and caffeine in the 24 h preceding the assessment, fasting for 3 h preceding the assessment, and minimum effort when traveling to the laboratory. On arrival to the laboratory, participants were asked to complete and sign a form to state whether they had conformed to these pre-test procedures. Participants then laid in a quiet environment for 10-min, after which the VO_2_ was measured for 40-min in the supine position. The average VO_2_ data between minutes 35–40 was used to calculate the %VO_2_R for prescription of the exercise bouts, since this time period has been previously shown to elicit a VO_2_ steady-state and high test–retest reliability (Cunha et al. 2013).

### Cardiopulmonary exercise testing and constant work rate exercise bouts

The CPET involved a ramp-incremented protocol performed on an electronically-braked cycle ergometer (SRM GmbH, Jülich, Germany) to determine VO_2max_, as described elsewhere (Cunha et al. 2015a). The test was considered maximal if the subject satisfied at least three of the four following criteria: (a) maximum voluntary exhaustion as reflected by a score of 19 on the Borg 6–20 scale; (b) 90% of the predicted maximal heart rate (HR_max_ = 220 – age), or presence of a heart rate plateau (ΔHR between two consecutive work rates ≤ 4 beats/min); (c) presence of a VO_2_ plateau (ΔVO_2_ between two consecutive work rates < 2.1 mL kg/min^1^); and (d) a maximal respiratory exchange ratio (RER_max_) > 1.10 (Howley et al. 1995). All participants were verbally encouraged to provide a maximal effort.

To determine the work rate for the two exercise bouts, 60% VO_2_R was calculated from the VO_2max_ and resting VO_2_ and the obtained VO_2_ value was used to calculate the associated cycling power by applying the equation: VO_2_ cycling = 3.5 + 12.24 × power × (BW^−1^), where VO_2_ is in mL kg/min^1^, and power is in W and BW (body weight) is in kilograms (ACSM 2018). Cycling cadence was maintained at 70 (± 5) revs/min^1^ throughout the test and the mean (SD) power output was 136 (36) W. Net energy expenditure was calculated individually from the net VO_2_, which is the additional VO_2_ above resting VO_2_ induced by the exercise bout (i.e. net VO_2_ = gross VO_2_ − resting VO_2_) (Swain 2000). The net VO_2_ values expressed in mL/kg/min were converted to L/min^1^ and then to kcal/min. Based on the values obtained for VO_2_ and energy expenditure, the exercise bouts were terminated when each subject had achieved a total energy expenditure of 300 kcal. To negate the confounding effects of the initial (fast) VO_2_ on-kinetics (i.e. the anaerobic energy expenditure component of total energy expenditure), the first 5-min interval of each exercise bout was omitted from all analyses (Cunha et al. 2011a, b). Minute ventilation and pulmonary gas exchange were measured breath-by-breath using a silicone oro-nasal face mask connected to a metabolic cart (Oxycon Pro®, Jaegger, Hocksberg, Germany). The turbine flow-meter and gas analyzers of the metabolic cart were calibrated immediately before each exercise test according to the manufacturer's instructions using a 3-L calibration syringe (Quinton Instruments, Seattle, WA), ambient air, and certified standard gases containing 16.0 ± 0.02% oxygen and 5.0 ± 0.02% carbon dioxide (Cryoservice Ltd, Worcester, UK). The calibrations also were performed immediately after the test as an extra level of quality assurance that the cart was still working within accepted parameters at the end of each test. Heart rate was measured continuously using a telemetric cardiotachometer (RS800cx, Polar™, Kempele, Finland).

### Measurement at baseline and recovery period to experimental trials

#### Blood pressure and cardiac autonomic control responses

SBP, DBP and MAP, heart rate variability (HRV) and spontaneous baroreflex sensitivity (SBR) were derived by finger photoplethysmography with a height correction applied from a blood pressure recording from the left brachial artery (Finometer™, FMS, Amsterdam, The Netherlands). A cuff was placed on the left middle finger for a beat-by-beat blood pressure assessment. The data from the Finometer were downloaded onto a PC and analyzed by BeatScope Software (BeatScope 1.1a; Finapres Medical Systems, Amsterdam, The Netherlands). The BeatScope software performs beat-to-beat analysis of the finger arterial pressure and uses filtering and level correction to calculate reconstructed brachial pressures from finger pressures.

For spectral analysis of R–R interval time series, data were processed using a Fast Fourier Transform with Welch’s method, with a Hanning window with 50% overlap using a customized algorithm from HeartScope™ II software (version 1.4, A.M.P.S., LLC, New York, USA) (Badilini et al. 2005). Beat-by-beat R–R interval series were then converted into equally spaced time series with 256 ms intervals using cubic spline interpolation (Lazzoli et al. 2003; Task-Force 1996). Spectral analysis was expressed in normalized units (n.u.) (Task-Force 1996). The ratio between low frequency and high frequency bands (LF:HF) was used as an index of sympathovagal balance, with the LF band (0.04–0.15 Hz) being considered as a marker of sympathetic predominance, and the HF band (0.15–0.50 Hz) as a marker of parasympathetic predominance (Cohen and Taylor 2002). The BRS was analyzed from the alpha index from the low frequency band (α-LF) of the beat-by-beat SBP and pulse interval (Parati et al. 2000). Only detected spectral gains with coherence > 0.5 (arbitrary threshold) were accepted.

#### Hemodynamic responses

Q and SV were determined using an impedance cardiography device (PhysioFlow® Type PF05L1, Manatec, Macheren, France). SVR was calculated as the quotient between MAP and Q. The six ICG surface electrodes (FS-50, Skintact, Innsbruck, Austria) of the PhysioFlow® were placed on the subject’s dry skin as stipulated in the manufacturer’s instructions (two on the left side of the neck, one on the middle of the sternum, one on the rib bone closest to the left ventricle, and two along the center of the spine). Before attachment of the electrodes the skin was prepared to optimize signal quality. This involved shaving the skin with a disposal razor, cleaning with alcohol prep swabs (Barclay-Swann Ltd, Peterborough, UK), abrading with a special gel until the skin turns bright pink (Nuprep Skin Prep Gel, Weaver and Company, Aurora, CO), and then cleaning with water. After the electrodes and associated cables were attached, they were secured with micropore surgical tape and secured in place by subjects wearing a tight elasticated cotton vest (Colorline Surgifix® m 25, FRA® Production S.p.A, Italy). The PhysioFlow® auto-calibration procedure was started after obtaining a stable signal as detected by a color graph displayed on the screen by the device. The raw data of each subject was then downloaded onto a PC in Excel file format and reviewed for detection of non-physiological artifacts. These were values corresponding to SV values over time with differences of more than 20% of the mean SV during pre-exercise (average of the last 5-min before exercise) and postexercise (average every 10-min of recovery) that were concomitantly associated with an unchanged HR (i.e. differences of no more than 5% of the mean HR).

#### Thermoregulatory responses

Skin and core temperatures were measured continuously using a Squirrel Data Logger (2020 Series; Grant Instruments, Shepreth, UK). Participants self-inserted a rectal probe 10 cm past the anal sphincter for the measurement of core temperature (*T*_c_). Four calibrated surface thermistors were attached to the chest, thigh, leg, and arm on the right side of the body and secured in place using Transpore™ Surgical Tape (3 M Healthcare, St.Paul, MN, USA) for the determination of skin temperature (*T*_sk_) (Ramanathan 1964). Mean body temperature (MBT) was calculated using the following equation (Colin et al. 1971): *MBT *(in degrees Celsius) = 0.79 (*T*_c_) + 0.21 (*T*_sk_).

#### Plasma volume

Capillary blood samples were obtained from the fingertips for the determination of changes in PV derived from hemoglobin and hematocrit measurements (Dill and Costill 1974). Hemoglobin concentration was measured using a HemoCue Hb 201^+^ analyser (HemoCue AB, Ängelholm, Sweden). Hematocrit was determined by separating the blood cells and plasma by centrifuging ~100 μl of whole blood for 5-min at 3000 rpm (Hawksley MHC Centrifuge; Hawksley, Sussex, UK) and then measuring the hematocrit level using a Micro-Haemotocrit Reader (Hawksley, Sussex, UK).

#### Assessment of 21-h ambulatory blood pressure

Changes in blood pressure were assessed between 13:00 and 10:00 on the next day by a TM-2430 ambulatory blood pressure monitor (A&D Company Ltd, UK). The ABPM fulfilled the criteria of the British Hypertension Society protocol (O'Brien et al. 2000) and was auto-calibrated before each test according to the manufacturer’s instructions. The non-dominant arm was used for measurement using an appropriately sized cuff. Participants were instructed on how the device worked and when activated, to hold their arm as still as possible and also to proceed with normal activities, not to shower or exercise until the next morning. Beyond that, all of them were given a standardized activity diary to register during the 21-h monitoring period any unusual physical or emotional events, and sleep and wake times. The monitor was programmed to record SBP and DBP via oscillation every 15-min, except between the hours of 23:00 and 06:00, during which it recorded blood pressure every 30-min to minimize sleep disturbance. The display on the monitor was switched off to prevent feedback to participants. All blood pressure readings rejected by the TM-2430 software as being artifacts (e.g. readings > 250 mmHg) were excluded from analyses. Data sets with < 80% of blood pressure recordings present also were excluded from analyses. The ABPM was fitted to the participants after CON, TEMP and HOT for the next 21-h period. When each subject came back to the laboratory the data were downloaded to a computer for determination of average blood pressure for daytime, night, and waking hours.

### Statistics

All statistical analyses were completed using IBM SPSS Statistics 22 (SPSS Inc., Chicago, IL). Data were summarized using means and standard deviations (SD). Cohen's *d* effect sizes for mean differences were calculated and defined as small (0.20), moderate (0.50), and large (0.80) (Cohen 1988). Student *t* tests were used to investigate differences between VO_2_ and time to achieve 300 kcal throughout exercise, and postexercise changes in body weight and PV in TEMP vs. HOT, respectively. Absolute values for all physiological responses during the 60-min post-exercise period (using 6 × 10 min time bins) for each condition (CON, TEMP, and HOT) were investigated using linear mixed models. Random effects were included in models if they significantly improved model fit, as indicated by a likelihood ratio test. The linear mixed model for the LF:HF ratio exhibited heteroscedastic residuals, which was corrected using a natural log transform. Separate models were used to investigate mean differences in baseline physiological values and 21-h blood pressure responses between conditions. In the event of significant main effects or interaction effects post hoc pairwise comparisons, with Sidak-adjusted *P* values, were conducted. The relationship between delta PV and delta MAP in the 60-min postexercise recovery period in TEMP and HOT was investigated using the Pearson correlation coefficient. Statistical significance for all null hypothesis significance tests was regarded as *P* < 0.05.

## Results

Table 1 presents the mean (SD) VO_2_, time to achieve an energy expenditure of 300 kcal, and changes in body weight and PV during continuous exercise bouts at 60% VO_2_R in TEMP and HOT. Compared to TEMP, VO_2_ was significantly lower in HOT (mean difference = 0.16 L/min, *P* = 0.023), whereas the observed time to achieve an energy expenditure of 300 kcal at 60% VO_2_R was 11.9% higher in HOT versus TEMP (*P* = 0.014). Reductions in body weight and percentage plasma volume from baseline and post control to 60-min postexercise recovery were greater in HOT versus TEMP (*P* ≤ 0.01).

**Table 1:** Mean ± SD VO_2_, time to achieve 300 kcal energy expenditure, and changes in body mass and plasma volume from continuous exercise bouts in TEMP and HOT (*N* = 7)

Variables	TEMP	HOT	Mean diff	95% CI	*t* test	*P * value	Effect size (Cohen's *d*)
	Mean (SD)	Mean (SD)					
VO_2_ (L/min)	1.79 (0.44)	1.63 (0.43)	0.16	0.03–0.29	3.0	0.023	0.40
Time to achieve 300 kcal (s)	2125 (549)	2366 (681)	241	70–412	3.5	0.014	0.42
∆ Body mass (kg)	− 0.6 (0.4)	− 1.6 (0.4)	1.0	0.6–1.3	6.2	0.001	2.7
∆ Plasma volume (%)	− 1.8 (1.7)	− 4.3 (1.5)	2.4	0.8–4.0	3.7	0.010	1.68

*VO*_*2*_ oxygen uptake; *∆ body mass* difference between postexercise vs. baseline, *∆ plasma volume* difference between average 60-min postexercise vs. postcontrol

### Blood pressure responses

**Fig. 1:**
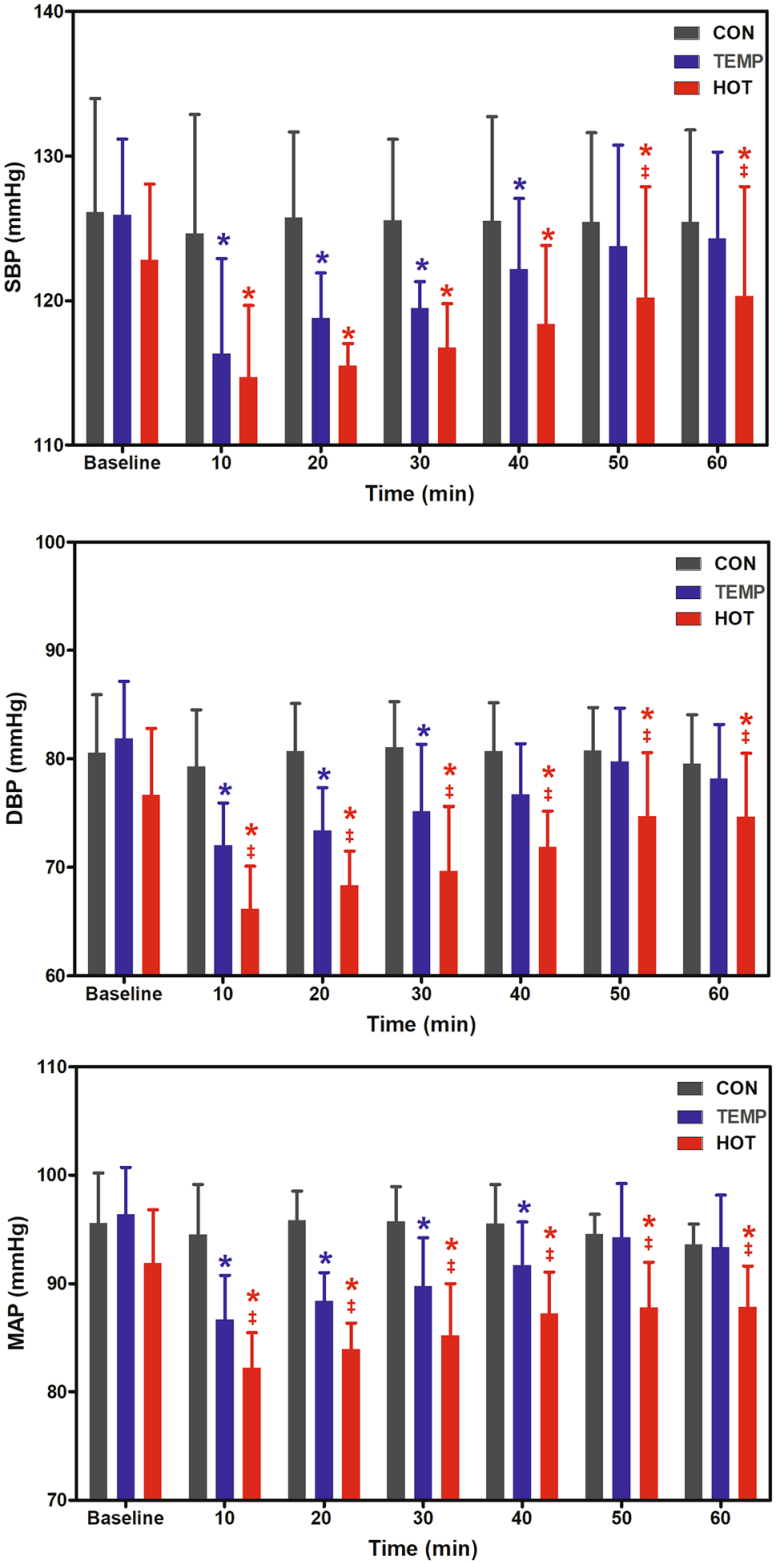
Systolic blood pressure (SBP), diastolic blood pressure (DBP), and mean arterial pressure (MAP) at baseline, during the 60 min control condition (CON), and the first 60 min of postexercise recovery in temperate (TEMP) and hot (HOT) conditions. *Significantly different to CON; ^‡^HOT significantly different to TEMP (*P* < 0.05)

**Fig. 2:**
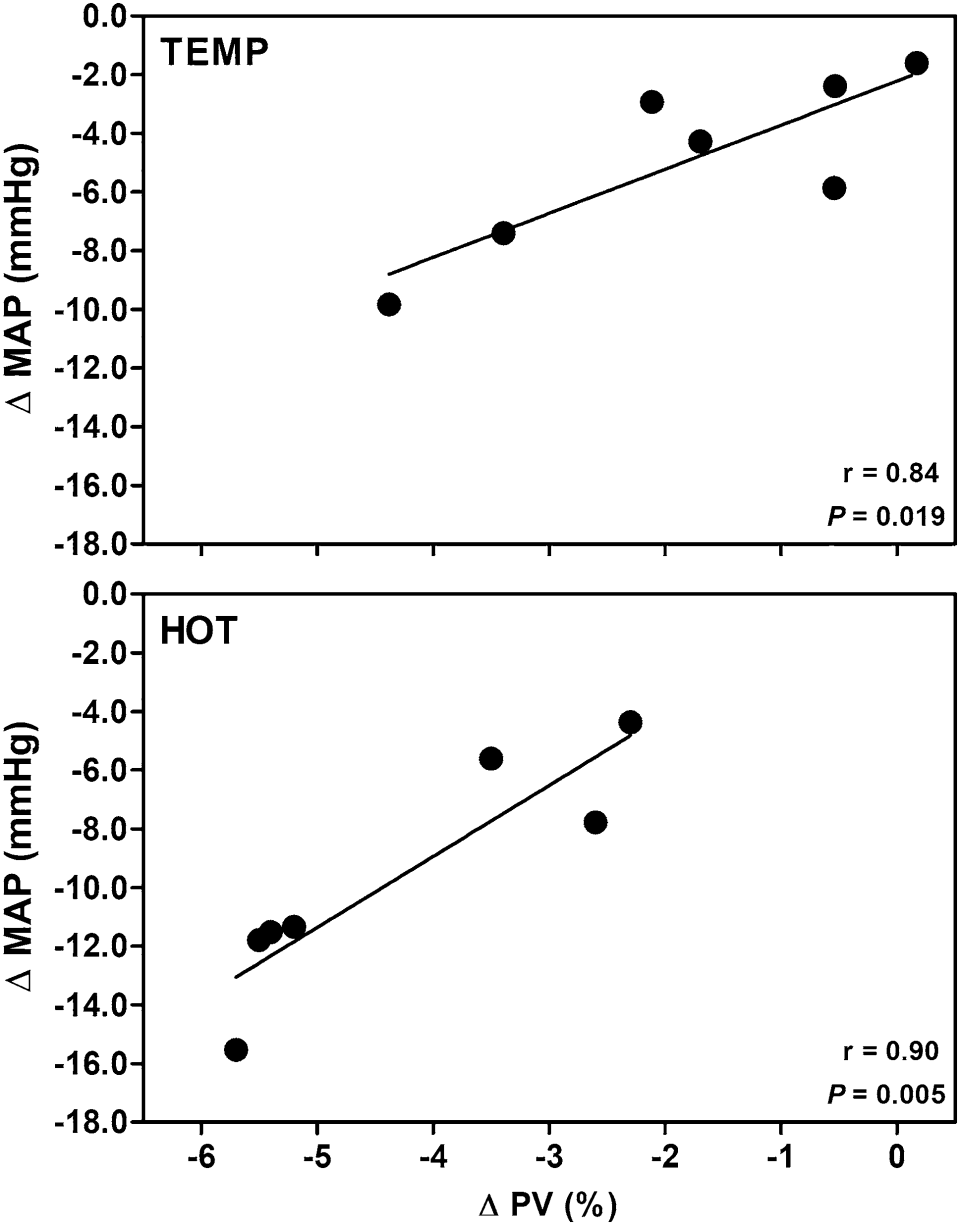
Relationship between the change in plasma volume (ΔPV) and change in mean arterial pressure (ΔMAP) during the first 60-min of postexercise recovery in the temperate (TEMP) and hot (HOT) conditions. Delta (Δ) = mean difference between CON and postexercise recovery

Figure 1 shows the blood pressure responses at baseline, during CON, and during the first hour of postexercise recovery in TEMP and HOT. Significant differences between conditions were observed, however, the differences were moderated by condition x time interactions for SBP (*F* = 12.4, *P* < 0.001), DBP (*F* = 9.6, *P* = 0.003), and MAP (*F* = 9.4, *P* < 0.001). Within 60-min post-CON, the coefficient of variation (CV) for SBP, DBP, and MAP were 5.0%, 5.2%, and 3.2%, respectively. The largest differences between CON and TEMP (SBP: 125 ± 7 and 122 ± 5 mmHg; DBP: 81 ± 4 and 77 ± 5 mmHg; MAP: 95 ± 4 and 91 ± 4 mmHg, respectively) and between CON and HOT (SBP: 125 ± 7 and 118 ± 5 mmHg; DBP: 81 ± 4 and 72 ± 3 mmHg; MAP: 95 ± 4 and 87 ± 4 mmHg, respectively) were observed in the first 40-min of recovery. Significant differences between TEMP and HOT were observed during the whole 60-min period for DBP and MAP, but only during the last 20-min for SBP. The changes in MAP over time were significantly related to the changes in PV in both the TEMP (*r* = 0.84, *P* = 0.019) and HOT (r = 0.90, *P* = 0.005) conditions (see Fig. 2).

### Hemodynamic responses

**Fig. 3:**
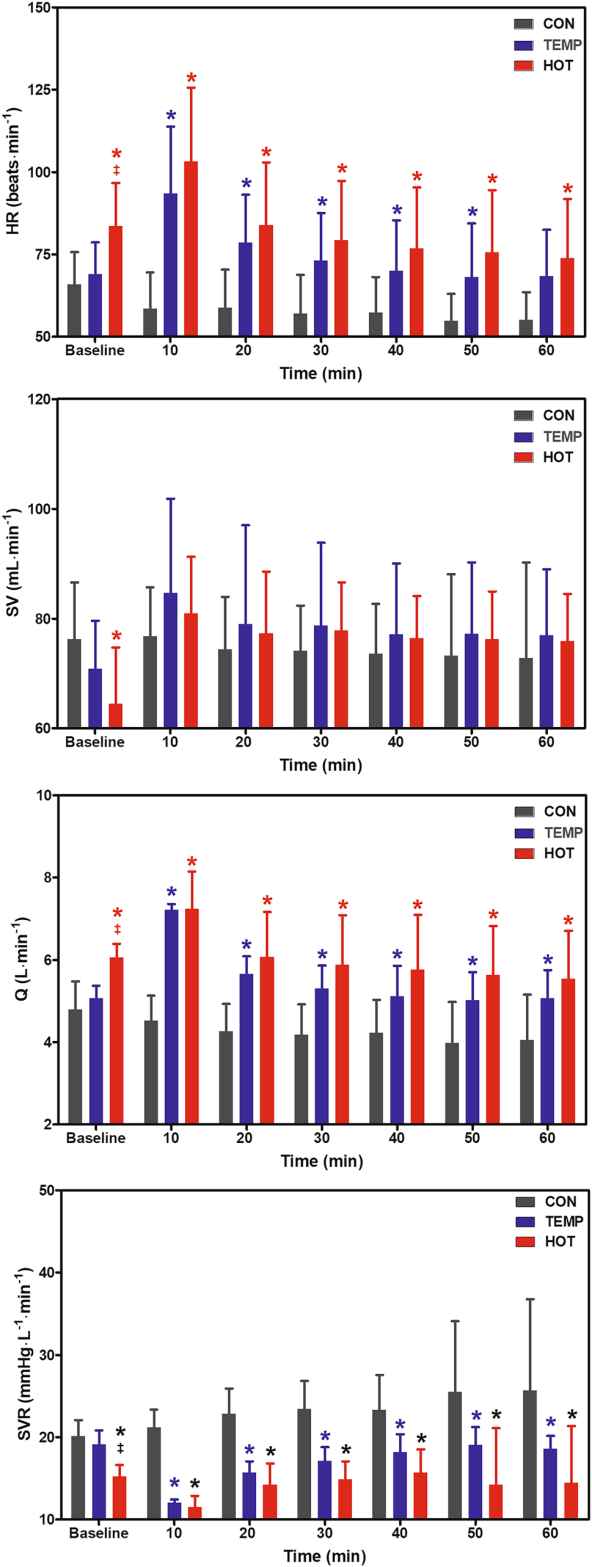
Heart rate (HR), stroke volume (SV), cardiac output (Q), and systemic vascular resistance (SVR) at baseline, during the 60 min control condition (CON), and the first 60 min of postexercise recovery in temperate (TEMP) and hot (HOT) conditions. *Significantly different to CON; ^‡^HOT significantly different to TEMP (*P* < 0.05)

Figure 3 shows the hemodynamic responses at baseline, during CON, and during the first hour of postexercise recovery in TEMP and HOT. The heart rate and Q were significantly higher in TEMP and HOT versus CON. The significant condition x time interactions for heart rate (*F* = 17.9, *P* < 0.001) and Q (*F* = 7.1, *P* = 0.004), however, indicated that these differences were greatest in the first 10-min of recovery. No significant differences between conditions were observed for SV. Significant differences between conditions also were observed for SVR (*F* = 19.7, *P* < 0.001), however, there was insufficient statistical evidence to suggest the differences were moderated by time (*F* = 1.5, *P* = 0.23). The SVR was 6.4 mmHg/L/min lower in TEMP compared to CON (*P* = 0.002) and 8.2 mmHg/L/min lower in HOT compared to CON (*P* < 0.001). The 1.8 mmHg/L/min lower SVR in HOT compared to TEMP was not statistically significant (*P* = 0.51).

#### Thermoregulatory responses

**Fig. 4:**
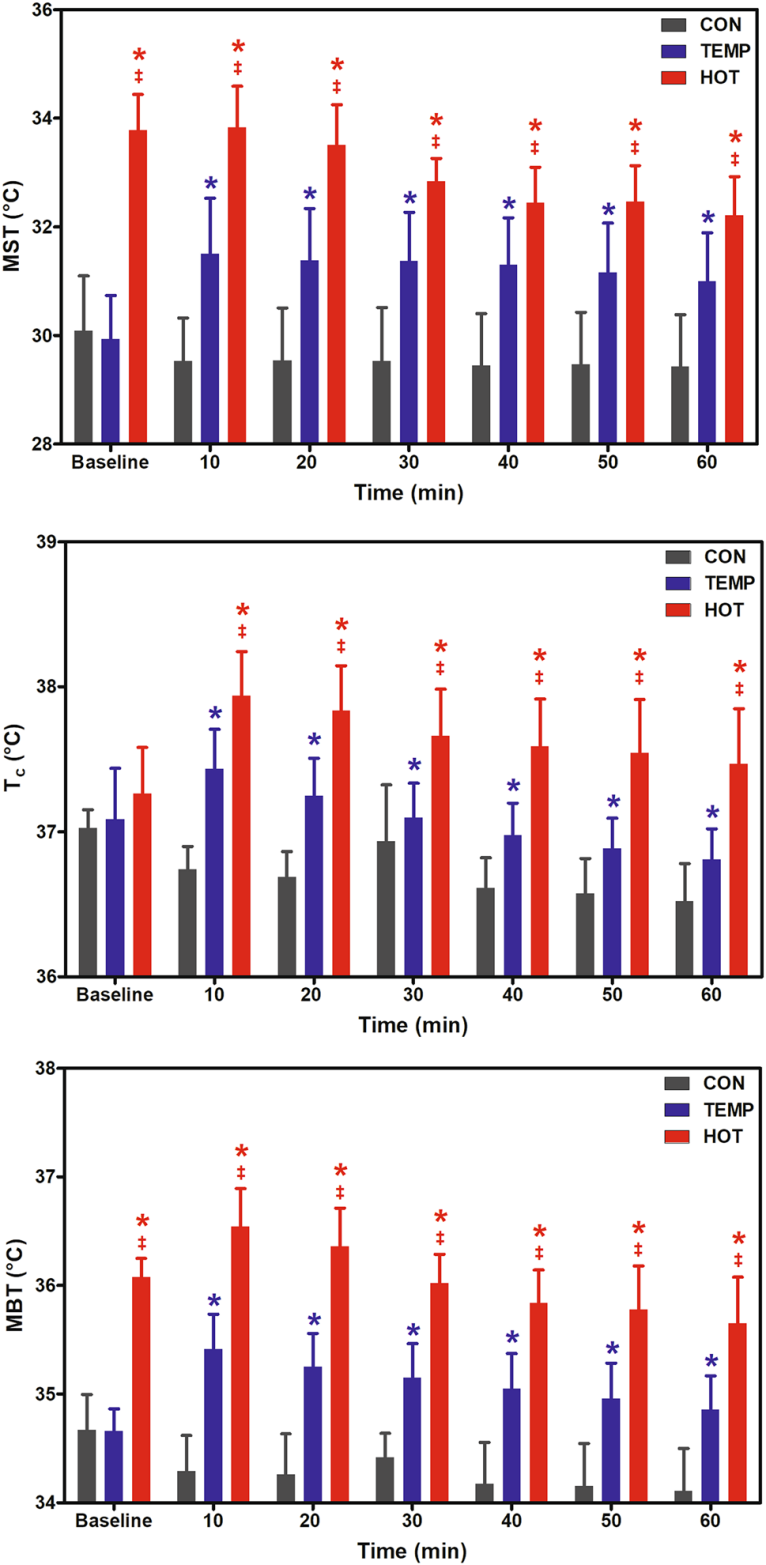
Mean skin temperature (MST), core temperature (*T*_c_), and mean body temperature (MBT) at baseline, during the 60 min control condition (CON), and the first 60 min of postexercise recovery in temperate (TEMP) and hot (HOT) conditions. *Significantly different to CON; ^‡^HOT significantly different to TEMP (*P* < 0.05)

Figure 4 shows the body temperatures at baseline, during CON, and during the first hour of postexercise recovery in TEMP and HOT. Significant differences between conditions were observed for mean skin temperature [MST (*F* = 112.9, *P* ≤ 0.001)], core temperature [*T*_c_ (*F* = 43.3, *P* =  < 0.001)], and mean body temperature [MBT (*F* = 198.9, *P* ≤ 0.001)], where body temperatures were significantly higher in TEMP and HOT compared to CON, and significantly higher in HOT versus TEMP. Significant condition × time interactions were observed for MST (*F* = 6.4, *P* = 0.002) and MBT (*F* = 7.2, *P* = 0.001), characterized by a significant difference in temperatures between HOT and CON and between HOT and TEMP during the 60-min of recovery.

### Cardiac autonomic responses

**Fig. 5:**
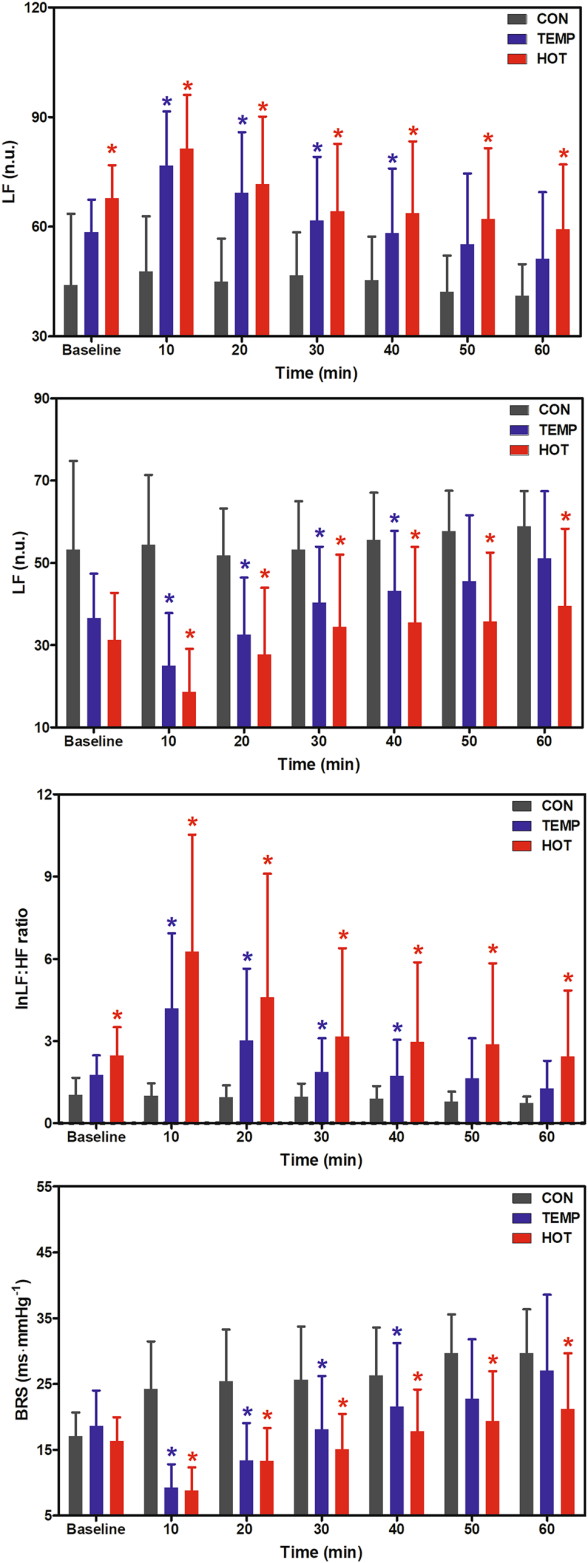
Low-frequency component (LF), high-frequency component (HF), logarithmically transformed sympatho-vagal balance (InLF:HF ratio), and baroreflex sensitivity (BRS) at baseline, during the 60 min control condition (CON), and the first 60 min of postexercise recovery in temperate (TEMP) and hot (HOT) conditions. *Significantly different to CON (*P* < 0.05)

Figure 5 shows the autonomic responses at baseline, during CON, and during the first hour of postexercise recovery in TEMP and HOT. Significant differences between conditions were observed for LF, HF, lnLF:HF ratio, and BRS. The LF and lnLF:HF ratio were higher, and HF and BRS lower, in TEMP and HOT compared to CON. Significant condition × time interaction effects for LF (*F* = 7.0, *P* = 0.004), HF (*F* = 7.2, *P* = 0.003), lnLF:HF ratio (*F* = 7.1, *P* = 0.005), and BRS (*F* = 16.3, *P* < 0.001), however, indicated that the greatest differences were observed during the first 30-min of recovery.

### 21-h ambulatory blood pressure responses

Table 2 shows the mean (SD) ambulatory SBP and DBP responses over the 21-h recovery period subsequent to the initial 1-h recovery. Significant differences in SBP between conditions were observed (*F* = 10.1, *P* = 0.006), where SBP was 4.2 mmHg lower in HOT compared to CON (*P* = 0.016) and 2.5 mmHg lower in HOT compared to TEMP (*P* = 0.039). There was no main effect for condition for DBP (*F* = 0.2, *P* = 0.80) and no condition × time interaction for either SBP (*F* = 0.9, *P* = 0.51) or DBP (*F* = 0.5, *P* = 0.72).

**Table 2:** Mean (SD) systolic and diastolic blood pressure during daytime (13:00–22:00 h), sleeping (23:00–06:00 h), and waking hours (06:00–10:00 h) in the control (CON), temperate (TEMP), and hot (HOT) conditions

	Systolic blood pressure (mmHg)						Diastolic blood pressure (mmHg)					
	CON		TEMP		HOT		CON		TEMP		HOT	
Day	123.5	(6.3)	120.7	(4.8)	118.0	(5.6)*	72.2	(5.0)	71.8	(1.5)	71.7	(2.7)
Sleep	105.5	(11.8)	105.5	(7.8)	100.8	(6.7)*	58.7	(7.8)	60.0	(8.7)	57.8	(6.6)
Awake	117.7	(6.5)	117.3	(12.2)	117.0	(3.8)*	70.2	(8.2)	65.0	(7.0)	68.8	(4.2)

^*^Significant main effect for condition (*P* < 0.05)

## Discussion

The present study adds to current knowledge by investigating whether an acute cycling bout performed in the heat prolongs hypotensive effects compared to a temperate environment in untrained men with elevated blood pressure. The main finding was that only the cycling bout expending 300 kcal at 60% VO_2_R in a hot environment (i.e. 35 °C) was able to induce statistically significant ambulatory SBP reductions during the 21-h observed recovery period, with the most prominent reductions occurring during the daytime (13:00 to 22:00 h) and sleeping (23:00–06:00 h) hours. Within this context, the physiological mechanisms that underpin the differences in PEH between HOT and TEMP (at least for the first 60-min recovery period) appear to be augmented core and skin temperatures, and a reduced PV via increased fluid loss, which may have led to persistent peripheral vasodilatation as the main pathway to evaporative heat loss following exercise in HOT.

To-date, few studies have investigated the influence of heat stress on PEH following aerobic exercise (Franklin et al. 1993; Lynn et al. 2009), and none investigated these responses in untrained men with elevated blood pressure using exercise bouts matched by energy expenditure, and none used ABPM. Only the exercise bout in the heat in our study was able to promote PEH over the 21-h recovery period subsequent to the initial 1-h recovery, since PEH was only evident for up to 40-min of the recovery period in TEMP (see Fig. 1 and Table 2). PEH can be considered a desirable effect of exercise, since chronic reductions in blood pressure from engaging in long-term aerobic exercise programs are due largely to the summative effects of the blunted blood pressure response observed after single acute bouts of aerobic exercise (Liu et al. 2011). It is worth noting, however, that more pronounced PEH such as that observed in the present study by ABPM following exercise in the HOT condition, increases the risk of vasovagal syncope, which is an undesirable response that may occur during recovery from exercise especially when performed in the heat (McCord et al. 2008). Indeed, caution must be taken regarding exercise prescription aimed at inducing PEH under heat stress conditions. Our findings showed that PEH over 22 h was substantially marked by reducing heat load through sweating, plasma volume shifts and vasodilatation. In places commonly characterized by temperatures higher than 35 °C (e.g. 40 °C in Rio de Janeiro, Brazil), however, it would be very likely that PEH could reflect marked dehydration, which would not be desirable among untrained subjects with elevated blood pressure and, therefore, not recommended for improving health.

Partially in disagreement with the results of a previous study (Franklin et al. 1993), since the present investigation was also able to observed PEH in TEMP, our findings indicates that augmented core and skin temperatures and decreased PV are the main hemodynamic mechanisms underlying a reduction in blood pressure after exercise performed under heat stress in untrained men. Compared to CON, PV was reduced by 1.8% and 4.3% during 60-min of recovery after TEMP and HOT, respectively. The greater reduction in PV in HOT, which was strongly associated with the reduction in MAP (see Fig. 2), was also due to greater fluid loss in HOT versus TEMP [e.g. body mass loss of 1.6 kg (2.1%) vs. 0.6 kg (0.8%), respectively] (see Table 1). This is in agreement with the findings of previous studies (Serwah and Marino 2006; Marino et al. 2010). Marino et al. (2010) observed a mean body mass loss of 1.61 kg (2.1%) in 7 moderately trained individuals (age: 20.6 ± 1.1 year; VO_2max_: 3.8 ± 0.2 L/min) in response to 60 min of intense self-paced cycling without hydration (6 × 1-min “all-out” sprints at 10-min intervals under ~ 33.2 °C and ∼ 64% relative humidity). Similarly, Serwah and Marino (2006) reported a decrease in body mass of 1.3 kg (1.7%) in eight moderately to highly trained men (age: 24.5 ± 1.0 years; VO_2max_: 3.6 to 4.7 L/min) after cycling for 90 min at ∼ 70% of peak power output in a warm condition (31 °C) without water replacement. Comparisons between the results of the aforementioned studies should be viewed with caution, however, considering the different exercise protocols, environmental conditions, baseline hydration level, the subject's tolerance to discomfort during heat exposure, and cardiorespiratory fitness status.

On the other hand, when compared to CON, HR was higher in HOT and TEMP (42% and 30%, respectively), which in turn induced a greater postexercise Q in both HOT and TEMP conditions (42% and 31%, respectively). Unlike heart rate and Q, SVR also was reduced by 29% and 50% during 60-min of recovery after TEMP and HOT, respectively (see, Fig. 3). Even in the absence of statistical significance between HOT and TEMP for such hemodynamic responses (i.e. likely a type II statistical error due to a small sample size), the SVR tended towards a persistent peripheral vasodilatation in HOT, which has been associated with elevations in skin and core temperature that induce changes in skin blood flow (Franklin et al. 1993). In this regard, the observations of Franklin et al. (1993) from 10 normotensive and highly fit endurance-trained men (age: ~ 21 years; SBP/DBP: ~ 121/63 mmHg; VO_2max_: ~ 4.73 ± 0.88 L/min) were somewhat similar to our results, if we consider changes in PEH in response to changes in environmental temperature. The authors demonstrated that the occurrence of the PEH phenomenon depended on the environmental condition in which the participants recovered from cycling in a temperate environment, compared to baseline. For example, a significant reduction in MAP of − 4.7 (~ 6%) mmHg was observed during 60-min recovery in the heat, but no changes were observed when the recovery took place in neutral and cool temperatures. Like MAP, significantly elevated skin and core temperatures were only observed for 60-min in the warm recovery condition, attenuating heat loss compared to neutral and cool conditions. According to the authors, taking into account that blood flow to the skin increases when core temperature and MST are elevated (Roddie and Shepherd 1956), a plausible explanation for the occurrence of PEH only in the warm condition was likely due to a persistent vasodilation (decreased SVR estimated in ~ 24%) in response to a significant elevation in skin and core temperatures for 60-min in the warm recovery condition (Franklin et al. 1993).

Lynn et al. (2009) investigated the PEH response to heat stress in 14 normotensive and highly fit endurance-trained men (age: ~ 26 years; SBP/DBP: ~ 111/61 mmHg; VO_2max_: 4.6 L/min) after performing 60-min cycling bouts at 60% VO_2peak_ under three conditions: (1) Control: temperate condition (22 °C and 30% humidity) without water replacement; (2) Fluid: temperate condition (22 °C and 30% humidity) with water replacement; and (3) Warm: hot condition (30 °C and 30% humidity) without water replacement. Notably, all exercise conditions elicited similar reductions in MAP during the 90-min recovery period compared to baseline (*P* > 0.05). Like MAP, postexercise Q and systemic vascular conductance in Control were lower than baseline (*P* = 0.001), while the Fluid condition partially mitigated the postexercise decrease in Q (e.g. ∼ 0.41 L/min higher than Control). Furthermore, exercise in the Warm condition also attenuated the decrease in postexercise Q, suggesting a role for thermal load on the haemodynamic recovery pattern from exercise performed in heat stress. Similarly to Q, postexercise systemic vascular conductance remained above baseline values for Fluid and Warm conditions. The authors hypothesized that this novel and unanticipated finding may be attributable to an elevated core temperature, which increased heart rate and myocardial contractility. However, results from the present study are not consistent with this premise, since the magnitude and duration of hypotension during the 22-h recovery from cycling bouts were significantly greater in HOT versus TEMP (see Fig. 1 and Table 2). These conflicting findings might be due to the characteristics of the volunteers, since Lynn et al. (2009) compared these responses in highly fit endurance-trained men compared to the present study that included untrained men with elevated blood pressure (mean VO_2max_: 2.80 L/min).

The PEH observed in both TEMP and HOT conditions was also followed by a significant reduction in BRS during the postexercise recovery compared to CON (see Fig. 5). Additionally, the HRV analysis showed that during recovery sympathetic activity (LF component) remained more elevated than parasympathetic activity (HF component), resulting in higher sympathovagal balance (LF:HF ratio) concomitant to the reduction in blood pressure (see Fig. 1). The data from the present study concur with previous reports from studies using spectral analysis (Cunha et al. 2015b, 2016; Fonseca et al. 2018; Halliwill et al. 1996a) indicating that PEH was accompanied by a shift in cardiac autonomic balance, characterized by increased sympathetic and reduced parasympathetic activity, where the attenuated BRS also suggests a persistent sympathetic rather than vagal activation after exercise. Furthermore, from our findings it might be speculated that such increases in sympathetic activity could be a physiological response to offset the reduction in SBP and to compensate for the resetting of the baroreflex (Halliwill et al. 1996b).

Some limitations of this study must be acknowledged. Firstly, it was not possible to control the physical activity levels during the 21-h recovery period assessed by accelerometry to ensure that the physiological demands during most of the recovery period were similar among CON, TEMP and HOT conditions. Secondly, for logistic reasons it was not possible to weigh the participants 22 h after the exercise bouts or control fluid intake post-exercise and, therefore, there is a possibility that changes in BP could be at least partly due to un-replenished fluid losses. Thirdly, the mean differences between CON and TEMP for SBP (∆-3 mmHg), DBP (∆-4 mmHg), and MAP (∆-4 mmHg) were below or similar to the CVs of 5% (6 mmHg), 5.2% (4 mmHg), and 3.2% (3 mmHg), respectively. However, this methodological issue was not observed in HOT condition, since the mean changes in the first 60-min of recovery for SBP (∆-7 mmHg), DBP (∆-9 mmHg) and MAP (∆-8 mmHg) were higher than those CVs related to post-CON. Fourthly, the sample size of 7 subjects was small and may have favored the type II error for the physiological variables investigated after exercise and our data must be interpreted with this in mind.

In conclusion, exercise in the heat increases the hypotensive effects following exercise for at least 22 h in untrained men with elevated blood pressure. The underlying mechanisms (at least for the first 60-min recovery period) appear to be increased core and skin temperatures, and a reduced PV via increased fluid loss, which may have led to persistent peripheral vasodilatation produced as the main pathway of heat loss following exercise in HOT. Further research is warranted to ratify the present findings, by observing PEH and its potential underlying mechanisms following exercise bouts in the heat, using different exercise modes and intensities matched for overall volume.

## Abbreviations

%VO_2_R: Percentage of oxygen uptake reserve
ABPM: Ambulatory blood pressure monitoring
BRS: Baroreflex sensitivity
CON: Non-exercise control session
CPET: Maximal cardiopulmonary exercise test
DBP: Diastolic blood pressure
HF: High frequency band
HOT: Hot environment
HR: Heart rate
HRR: Heart rate reserve
HRV: Heart rate variability
LF: Low-frequency band
LFHF ratio: Sympathovagal balance
MAP: Mean arterial pressure
MST: Mean skin temperature
PEH: Postexercise hypotension
PV: Plasma volume
Q: Cardiac output
RER: Respiratory exchange ratio
SBP: Systolic blood pressure
SV: Stroke volume
SVR: Systemic vascular resistance
*T*_c_: Core temperature
TEMP: Temperate environment
*T*_sk_: Skin temperature
VCO_2_: Carbon dioxide output
*V*_E_: Minute ventilation
VO_2_: Oxygen uptake
VO_2_R: Oxygen uptake reserve
